# Integrated bioinformatics analysis of core regulatory elements involved in keloid formation

**DOI:** 10.1186/s12920-021-01087-7

**Published:** 2021-10-02

**Authors:** Chuying Li, Meitong Jin, Yinli Luo, Zhehu Jin, Longquan Pi

**Affiliations:** Klebs Research Center, Department of Dermatology, Yanbian University Hospital, Yanji, 133000 China

**Keywords:** Keloids, Integrated bioinformatics analysis, MicroRNAs, Transcription factor

## Abstract

**Background:**

Keloid is a benign fibro-proliferative dermal tumor formed by an abnormal scarring response to injury and characterized by excessive collagen accumulation and invasive growth. The mechanism of keloid formation has not been fully elucidated, especially during abnormal scarring. Here, we investigated the regulatory genes, micro-RNAs (miRNAs) and transcription factors (TFs) that influence keloid development by comparing keloid and normal scar as well as keloid and normal skin.

**Methods:**

Gene expression profiles (GSE7890, GSE92566, GSE44270 and GSE3189) of 5 normal scar samples, 10 normal skin samples and 18 keloid samples from the Gene Expression Omnibus (GEO) database were interrogated. Differentially expressed genes (DEGs) were identified between keloid and normal skin samples as well as keloid and normal scar samples with R Project for Statistical Computing. Gene Ontology (GO) functional enrichment analysis was also performed with R software. DEG-associated protein–protein interaction (PPI) network was constructed by STRING, followed by module selection from the PPI network based on the MCODE analysis. Regulatory relationships between TF/miRNA and target genes were predicted with miRnet and cytoscape. Core regulatory genes were verified by RT-qPCR.

**Results:**

We identified 628 DEGs, of which 626 were up-regulated and 2 were down-regulated. Seven core genes [neuropeptide Y(*NPY*), 5-hydroxytryptamine receptor 1A(*HTR1A*), somatostatin (*SST*), adenylate cyclase 8 (*ADCY8*), neuromedin U receptor 1 (*NMUR1*), G protein subunit gamma 3 (*GNG3*), and G protein subunit gamma 13 (*GNG13*)] all belong to MCODE1 and were enriched in the “G protein coupled receptor signaling pathway” of the GO biological process category. Furthermore, nine core miRNAs (hsa-mir-124, hsa-let-7, hsa-mir-155, hsa-mir-26a, hsa-mir-941, hsa-mir-10b, hsa-mir-20, hsa-mir-31 and hsa-mir-372), and two core TFs (SP1 and TERT) were identified to play important roles in keloid formation. In the TF/miRNA-target gene network, both hsa-mir-372 and hsa-mir-20 had a regulatory effect on *GNG13*, *ADCY8* was predicted to be target by hsa-mir-10b, and *HTR1A* and *NPY* were potentially by SP1. Furthermore, the expression of core regulatory genes (*GNG13, ADCY8, HTR1A and NPY*) was validated in clinical samples.

**Conclusions:**

*GNG13, ADCY8, NPY and HTR1A* may act as core genes in keloid formation and these core genes establish relationship with SP1 and miRNA (hsa-mir-372, hsa-mir-20, hsa-mir-10b), which may influence multiple signaling pathways in the pathogenesis of keloid.

**Supplementary Information:**

The online version contains supplementary material available at 10.1186/s12920-021-01087-7.

## Background

Keloid is a morbid and unique fibro-proliferative dermal disorder formed by an abnormal scarring response to injury [1]. Different from normal scar, keloid possesses tumor analogous properties including invasive uncontrolled growth and frequent relapses [2]. The growth of keloid can last for months to years and often causes pain, itching, and even movement restrictions. Keloids are unaesthetic and often accompanied by a psychological burden that results in decreased quality of life [3].

Accumulating evidence indicates a genetic predisposition for keloid because its incidence is greater in twins, families, and Asian and African ethnicities [2]. Several studies have focused on genetic factors in the pathogenesis of keloid formation; however, no single genetic cause has been identified. Gene expression profiles in keloids have also been determined [4–6] and have identified some important differentially expressed genes (DEGs). Target networks have also been generated to identify target microRNAs (miRNAs) and transcription factors (TFs). Studies on miRNAs of keloid have documented certain effects on angiogenesis, extracellular matrix, apoptosis and proliferation [7–9]. TFs including FOXM1, RUNX2, STAT3, YAP and SFRP1 have also been confirmed to be involved in the keloid [10–13]. However, most studies compared keloid with normal skin and not with normal scar tissue.

Keloids are pathological scars and comparison with normal scar tissue is necessary for a thorough understanding of keloid pathogenesis. In this study, to effectively identify DEGs differentially expressed genes, we compared keloid with normal skin and normal scar. The intersecting DEGs from these two comparisons were used for subsequent analysis. In addition, we merged four datasets by integrated bioinformatics methods to expand our sample size. We established a regulatory network to predict miRNAs and TFs as up-stream regulators of the common DEGs. This provides a basis for improved understanding of molecular basis of keloid pathology and effective pharmaceutical targets.

## Methods

### Data resources

GSE7890, GSE92566, GSE44270 and GSE3189 mRNA expression profiles (Homo sapiens) were downloaded from the Gene Expression Omnibus (GEO) database. Ten samples (five keloid and five normal scars) from GSE7890, twelve samples (nine keloid and three normal skin) from GSE44270, four keloid samples from GSE92566, and seven normal skin samples were from GSE3189. The expression profiling had been conducted using various Affymetrix platforms as follows: GSE3189 and GSE44270 belonged to GPL96 (Affymetrix Human Genome U133A) and GPL6244 (Affymetrix Human Gene 1.0 ST Array), respectively. GSE92566 and GSE7890 belonged to GPL570 (Affymetrix Human Genome U133 Plus 2.0 Array).

### Data preparation and screening for DEGs

Downloaded platform files and series matrix were converted into gene symbols usage of the limma package in R (V4.0.0) (https://www.bi-oconductor.org/packages/release/bioc/html/limma.html)and the following procedures run: conversion of gene ID, merging datasets, analysis of potential batch effects, data normalization and calculation of gene expression. The average values of probes were taken as the final expression of the genes that multiple probes matching. Screening of DEGs was then performed using the R package-limma. Three groups of samples were assessed: normal scar samples from adults (n = 5), normal skin samples from individuals who underwent surgery (n = 10) and keloid samples from resection surgery (n = 18). To identify as many genes related to the pathogenesis of keloids as possible, keloid samples were compared with normal scar and normal skin samples. The common DEGs were identified with online Venn diagram tools (http://bioinfogp.cnb.csic.es./tools/venny/index.html and http://bioinformatics.psb.ugent.be/webtools/Venn/). The DEGs depicted on the volcano plot were selected using filter conditions of *p*-value < 0.05 and |log2 fold change (log_2_FC)|> 1.

### GO Functional enrichment analysis

A GO functional enrichment analysis of DEGs was performed using the cluster Profiler package in R. Terms were assigned under three GO categories: biological process(BP), molecular function(MF), and cellular component(CC) [11]. We used ClueGO (v2.5.5), a Cytoscape plug-in [12], to group the GO terms for further analysis.

### Protein–protein interaction (PPI) network analysis and subnet module analysis

Search Tool for the Retrieval of Interacting Genes/Proteins (STRING; http://string-db.org/) was used to construct interaction networks among the DEGs-coded proteins(setting criteria: medium confidence ≥ 0.9) [13]. The most significant PPIs were plotted with the application of Cytoscape (v3.7.2, http://cytoscape.org/) [14] and the top module with the highest score (setting: k-core > 2) was identified. The proteins encoded by genes in the same module tended to have the same or analogous functions, and these proteins were enriched for the same biological role [15]. The employment of MCODE algorithm was used to evaluate the score of each module, a high score indicated enrichments and closer interactions [16]. To screen for the top hub genes, the PPI networks were analyzed by “cytohubba” plug-in in cytoscape software with two algorithm methods, including Maximal Chique Centrality (MCC) and degree methods using the MCODE plug-in.

### Prediction of networks mutually regulated by miRNAs and TFs.

To detect target genes that may be regulated by both miRNAs and TFs, we used miRnet, a network-based visual analysis tool, and the data in miRTarBase, a database of miRNA-target interactions, and miRecords, a resource for miRNA-target interaction. The highest involvement of miRNAs and TFs in a network was determined using-cytoHubba, a Cytoscape plug-in.

### Reverse transcription-quantitative ploymerase chain reaction (RT- qPCR)

We validated the expression pattern of hub genes by RT-qPCR. We collected 10 keloid and 10 normal skin tissue samples from the Dermatology Department of Yanbian University Hospital. Ethical approval for the study was granted by the Yanbian University Hospital Committee (Approval ID: 2018209) and written informed consent was obtained from the participating patients. Total RNA was extracted using TRIzol reagent (Invitrogen, Carlsbad, CA, USA) and complementary DNA was generated using a reverse transcription kit (Qiagen, Valencia, CA, USA) following the manufacturer’s instructions. RT-qPCR was performed using a miScript SYBR Green PCR kit (Qiagen) on an ABI 7300 real-time quantitative PCR system to obtain the expression levels of target genes. The reactions were performed in triplicate and average transcription levels were determined and normalized to corresponding GAPDH expression levels as an internal control. Relative mRNA levels were calculated using the delta-delta Ct method. Statistically significant differences are indicated by *. **p < 0.01, ***p < 0.001.

## Results

### Selection of DEGs

**Fig. 1:**
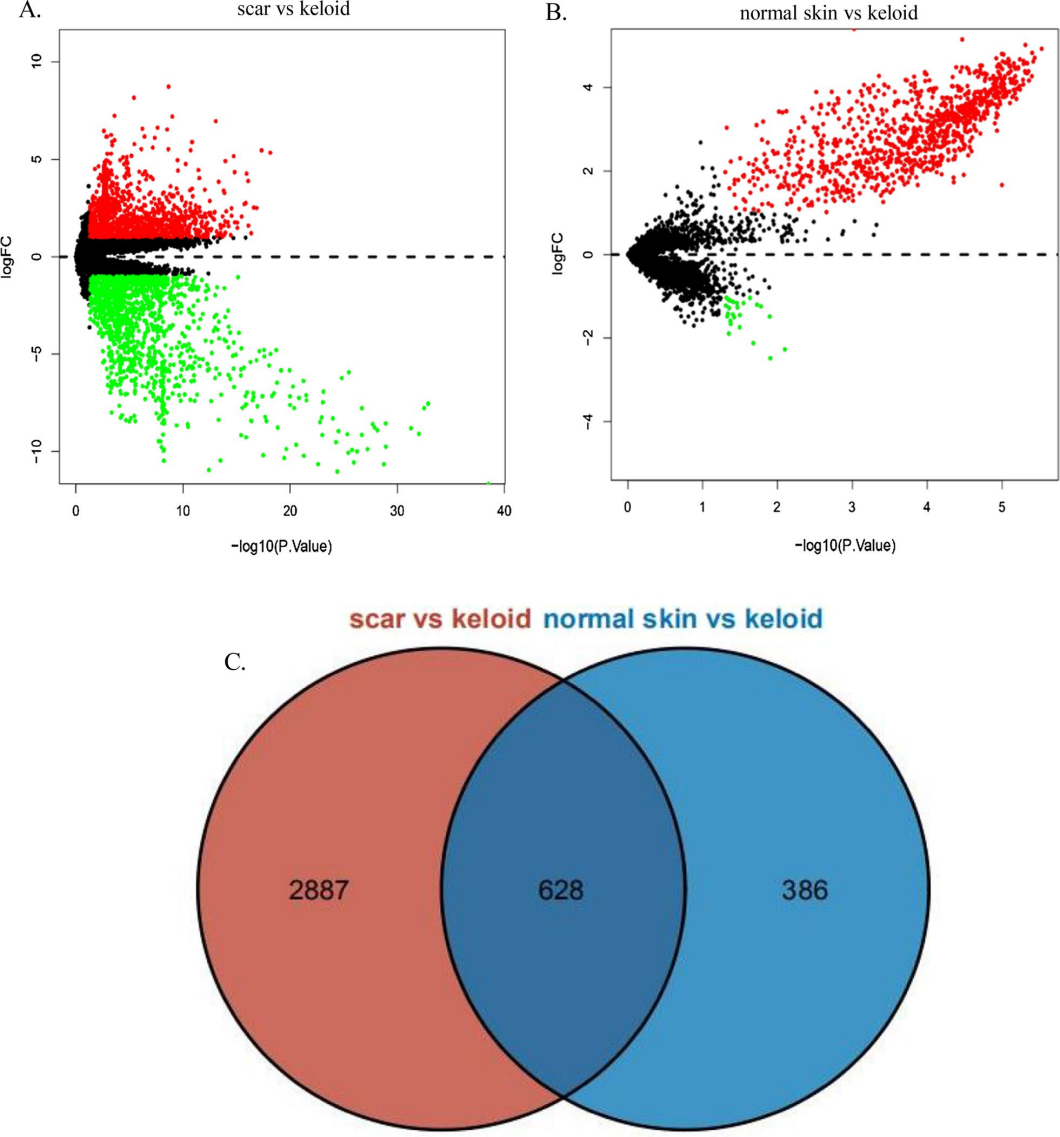
Identification of DEGs (*P* < 0.05, |log2FC|> 1). **A** A volcano plot of DEGs between keloid samples and normal scar samples. **B** A volcano plot of DEGs between keloid samples and normal skin samples. **C** Venn diagrams of overlapping DEGs. DEGs: Differentially Expressed Genes

Genes were defined as differentially expressed at |log_2_FC|> 1 and *p* < 0.05 (Fig. 1A, B). The total number of 3901 DEGs were identified in keloid. There were 3515 DEGs in the comparison of keloid and normal scar and 1014 DEGs in the comparison of keloid and normal skin, as presented in a volcano plot. The 628 DEGs that overlap between the two sets are presented in a Venn diagram (Fig. 1C).

### GO functional enrichment analysis

**Fig. 2:**
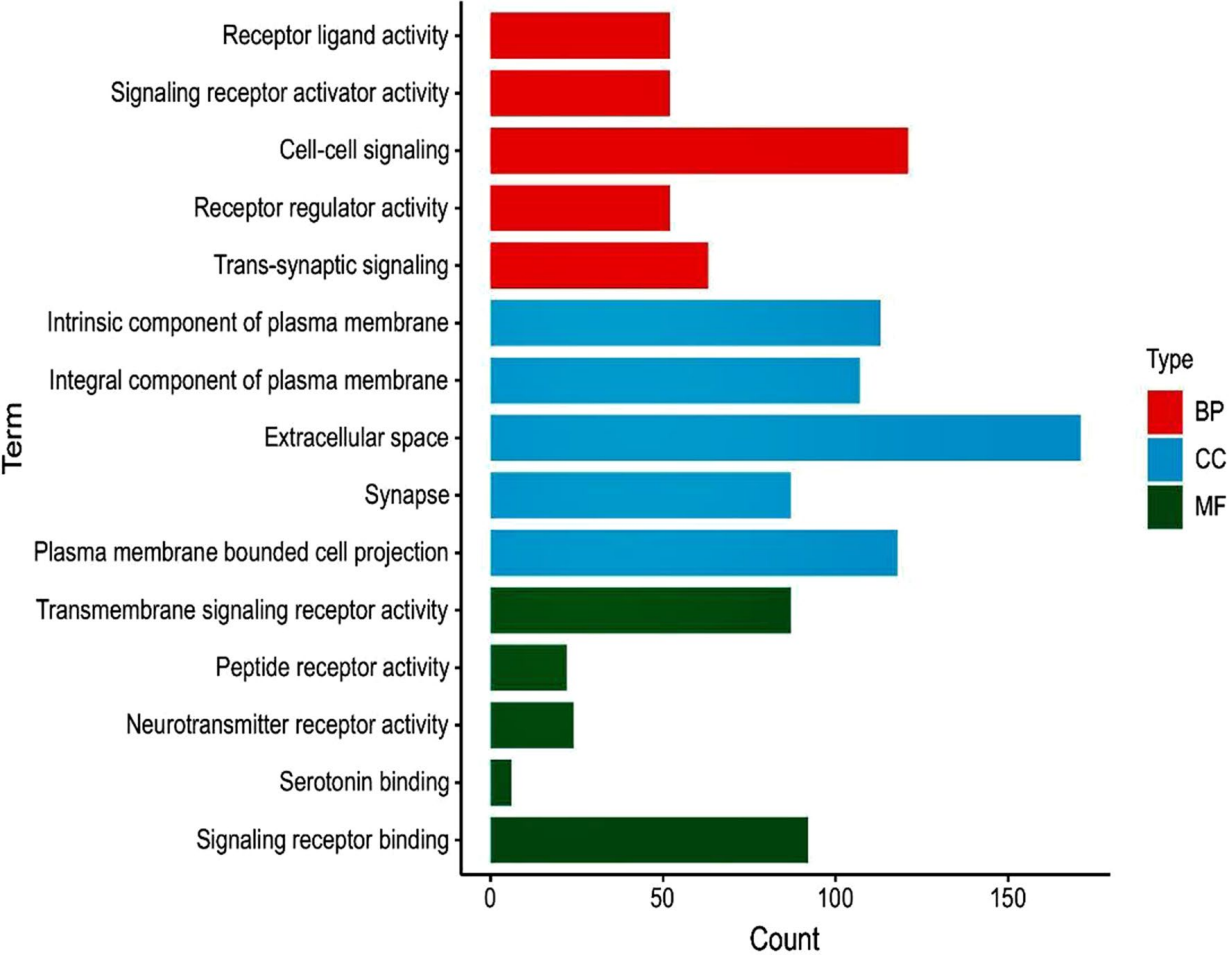
GO analysis of the common DEGs. GO, gene ontology

GO function analysis(*p*-value < 0.05) (Fig. 2, Table 1) revealed the most significant three terms belonging to BP, MF CC. For GO_BP, the DEGs were significantly enriched in signaling receptor ligand activity (*p*-value = 2.75E−10, counts = 52), signaling receptor activator activity (*p*-value = 4.27E−10, counts = 52), cell–cell signaling (*p*-value = 1.73E−09, counts = 121), receptor regulator activity (*p*-value = 9.93E−09, counts = 52), and trans-synaptic signaling (*p*-value = 1.19E−08, counts = 63). For GO_MF, DEGs were significantly enriched in intrinsic component of plasma membrane (*p*-value = 1.69–10, counts = 113), integral component of plasma membrane (*p*-value = 1.43–09, counts = 107), extracellular space (*p*-value = 1.44E−06, counts = 171), synapse (*p*-value = 5.09E−06, counts = 87), and plasma membrane bounded cell projection (*p-*value = 9.54E−05, counts = 118). GO_CC was enriched in transmembrane signaling receptor activity (*p*-value = 8.35E−07, counts = 87), peptide receptor activity (*p*-value = 5.07E−06, counts = 22), neurotransmitter receptor activity (*p*-value = 3.5E−05, counts = 24), serotonin binding (*p*-value = 0.000406, counts = 6), and signaling receptor binding (*p*-value = 0.001698, counts = 92).

**Table 1:** The top five results of GO analysis of the common DEGs ranked by *p* value

GO/ID	Term	*p* value	Count
*GO/BP*			
GO:0048018	Receptor ligand activity	2.75303E−10	52
GO:0030546	Signaling receptor activator activity	4.27219E−10	52
GO:0007267	Cell–cell signaling	1.73456E−09	121
GO:0030545	Receptor regulator activity	9.92932E−09	52
GO:0099537	Trans-synaptic signaling	1.19345E−08	63
*GO/CC*			
GO:0031226	Intirnsic component of plasma membrane	1.68629E−10	113
GO:0005887	Integral component of plasma membrane	1.43371E−09	107
GO:0005615	Extracellular space	1.44488E−06	171
GO:0045202	Synapse	5.0892E−06	87
GO:0120025	Plasma membrane bounded cell projection	9.53943E−05	118
*GO/MF*			
GO:0004888	Transmembrane signaling receptor activity	8.35E−07	87
GO:0001653	Peptide receptor activity	5.07E−06	22
GO:0030594	Neurotransmitter receptor activity	3.5E−05	24
GO:00051378	Serotonin binding	0.000406	6
GO:0005102	Signaling receptor binding	0.001698	92

### Analysis of hub genes and modules in the PPI network

**Fig. 3:**
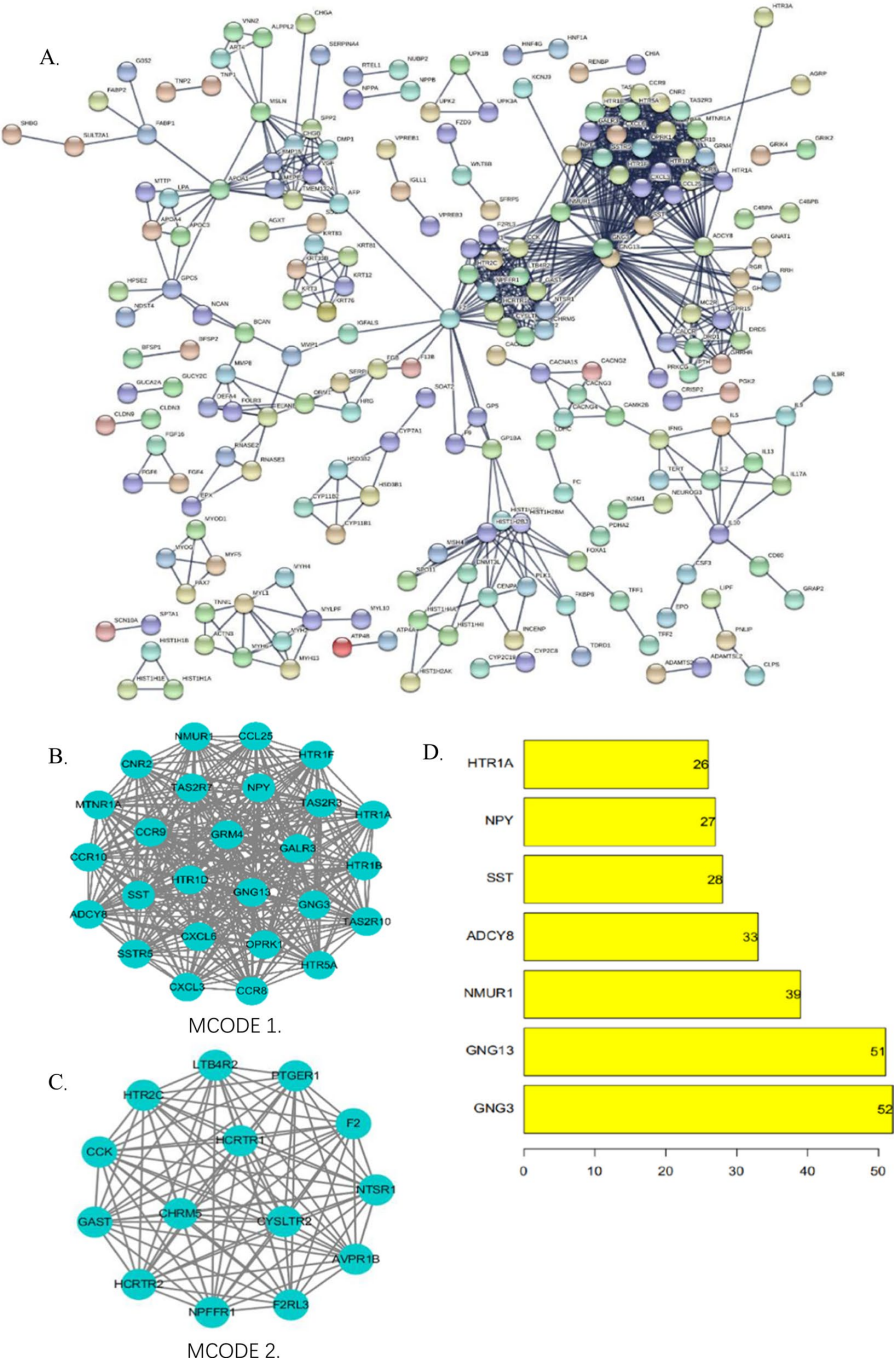
Identification of hub genes and analysis of the significant modules from PPI network. **A** The PPI network of DEGs was constructed using Cytoscape. **B**, **C** The significant module was obtained from PPI network. **D** The seven hub genes in the PPI network. PPI: Protein–Protein Interaction; DEGs: Differentially expressed genes

The PPI network of the DEGs was established using the STRING database and visualized with R. The network had 217 nodes and 1586 edges (Fig. 3A). To further screen for hub genes, we combined Maximal Chique Centrality (MCC) and degree method, to re-screen DEGs (Additional files 1, 2). The top seven genes with degree > 25 and the highest MCC affiliated to PPI were identified and included, neuropeptide Y (*NPY*) (degree = 27), 5-hydroxytryptamine receptor 1A (*HTR1A*) (degree = 26), somatostatin (*SST*) (degree = 28), adenylate cyclase 8 (*ADCY8*) (degree = 33), neuromedin U receptor 1 (*NMUR1*) (degree = 39), G protein subunit gamma 13 (*GNG13*) (degree = 51), G protein subunit gamma 3 (*GNG3*) (degree = 52) (Fig. 3D). We also screened functional subset-modules MCODE1 to MCODE2 for the PPI network (setting criteria: K-core > 2, MCODE SCORE ≥ 3 and network nodes > 4). We found that both MCODE1 and MCODE2 comprised up-regulated genes (Fig. 3B, C).Thirteen nodes (ADCY8, CNR2, GALR3, GRM4, HTR1A, HTR1B, HTR1D, HTR1F, HTR5A, MTNR1A, NPY, OPRK1, and SSTR5) of MCODE1 were enriched in the “G protein-coupled receptor signaling pathway” (GO:0007187). Eight nodes (AVPR1B, CYSLTR2, F2RL3, HCRTR1, HCRTR2, LTB4R2, NPFFR1, and NTSR1) of MCODE2 were enriched in the “G protein-coupled receptor signaling pathway, coupled to cyclic nucleotide second messenger” (GO:0008528) and “peptide receptor activity” (GO:0001653).

### Identification of miRNA-targets and TF-targets interaction network

**Fig. 4:**
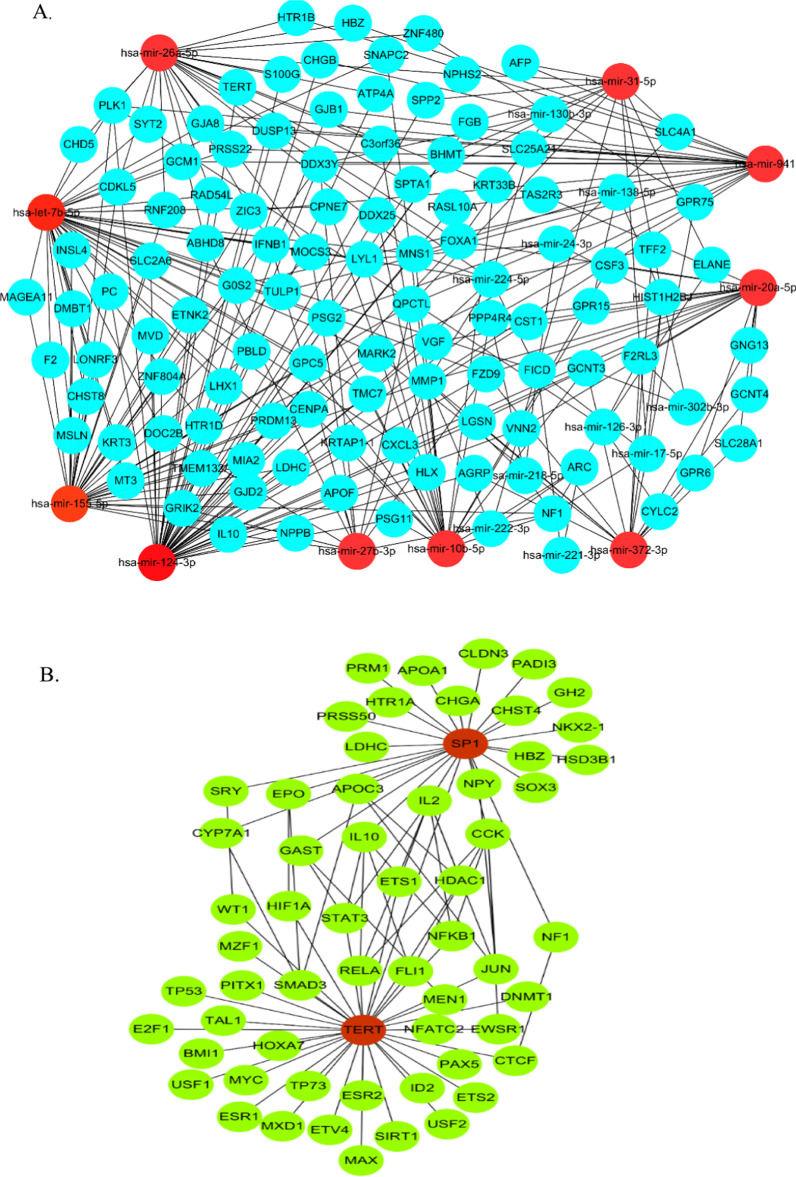
Network of miRNAs, TFs and target genes associated in keloid. **A** miRNAs-target regulatory networks. The red circle represents core miRNAs, and the blue circle represents the common DEGs. **B** TFs-target regulatory networks. The red circle represents core TFs, and the green circle represents the common DEGs. TFs: Transcription Factors; DEGs: Differentially Expressed Genes

**Fig. 5:**
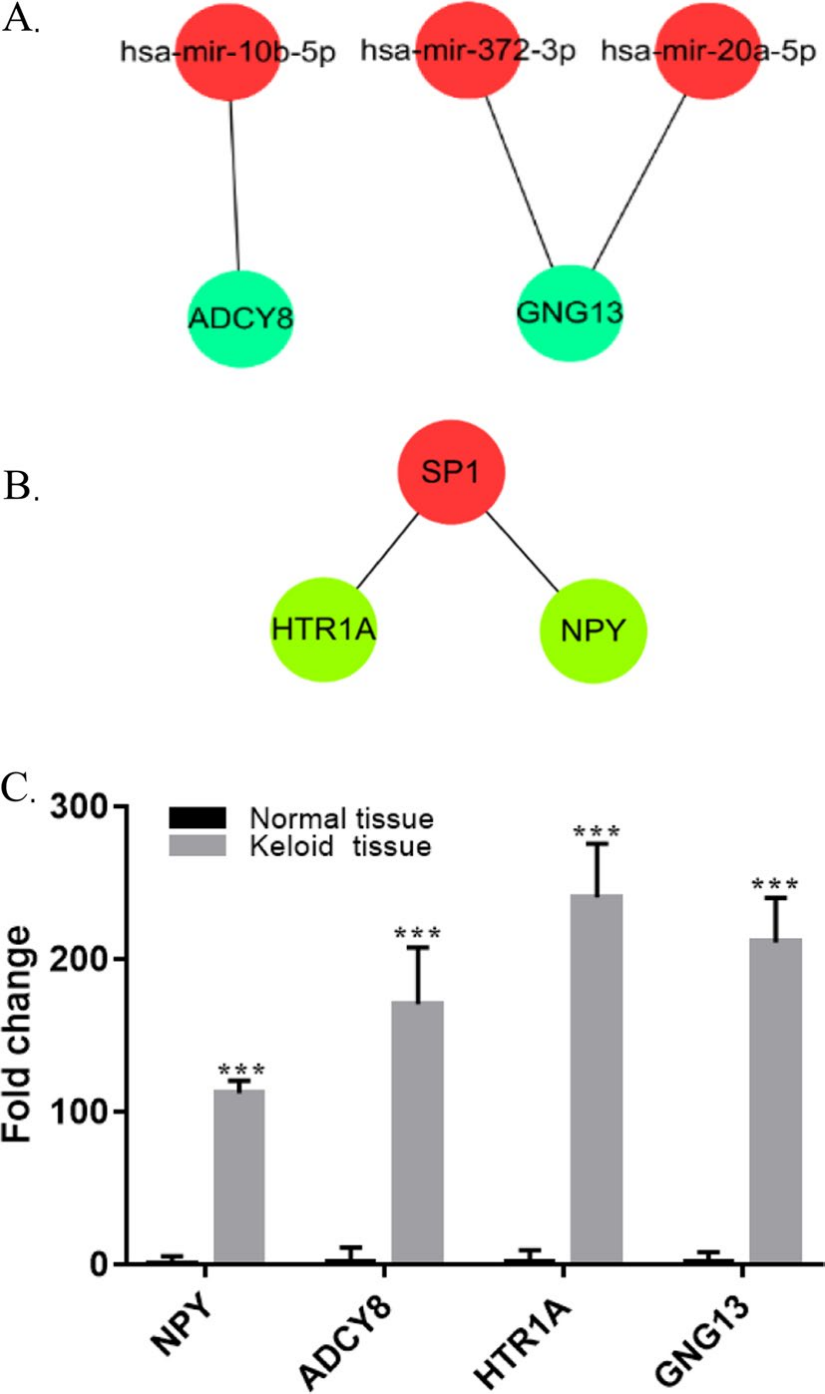
Analysis of crucial core regulative genes in keloid. **A** Schematic diagram of potential miRNAs-genes networks. **B** Schematic diagram of potential TFs-genes network. **C** RT-qPCR analysis of crucial core regulative genes. TFs: Transcription Factors

To understand the possible correlations between miRNAs and TFs using miRnet tool for DEGs. The miRNA-DEGs network contained 349 nodes and 648 edges (Additional file 3), and was analyzed further using MCC and degree methods. Hsa-mir-124, hsa-let-7, hsa-mir-155, hsa-mir-26a, hsa-mir-941, hsa-mir-10b, hsa-mir-20, hsa-mir-31 and hsa-mir-372 were all at least 25 degrees (Fig. 4A). Interestingly, the same results were obtained using the MCC method. Meanwhile, the TF-DEGs network (378 nodes and 622 edges) (Additional file 4) was also analyzed using the same algorithm and the two hub TFs, SP1 and TERT (Fig. 4B). We found a connection between the main up-regulators and hub genes. *GNG13* was a possible down-stream of hsa-mir-372 and hsa-mir-20, while *ADCY8* was predicted to be regulated by hsa-mir-10b (Fig. 5A). *HTR1A* and *NPY* were possibly regulated by SP1 (Fig. 5B). We observed no other significant up-stream regulators connected with remaining hub genes, including *SST*, *ADCY8* and *GNG3*.

### RT-qPCR

We narrowed down the number of hub genes by seeking connection between the main up-regulators and hub genes and validating by RT-qPCR. The mRNA levels of *NPY, ADCY8, GNG13* and *HTR1A* were significantly augmented in keloid compared with normal skin samples ***p < 0.001 (Fig. 5C).

## Discussion

We identified 628 DEGs (2 down-regulated and 626 up-regulated) by comparing keloid samples with normal scar and normal skin samples. The top seven important genes (*NPY, HTR1A, SST, ADCY8, NMUR1, GNG3 and GNG13*), all belong to MCODE1 and were enriched in the “G protein coupled receptor signaling pathway” GO_BP process. Furthermore, the most significant nine miRNAs (hsa-mir-124, hsa-let-7, hsa-mir-155, hsa-mir-26a, hsa-mir-941, hsa-mir-10b, hsa-mir-20, hsa-mir-31 and hsa-mir-372), and the top two TFs (SP1 and TERT) that play an important role in keloid formation were identified. In the TF/miRNA target gene network, *GNG13* was predicted to be targeted by hsa-mir-372 and hsa-mir-20, hsa-mir-10b had regulatory relationship with the *ADCY8*, and both HTR1A and NPY were potentially by SP1. Furthermore, the expression of core regulatory genes (i.e., *GNG13, ADCY8, HTR1A and NPY*) was validated in the clinical samples.

Keloids are benign fibrotic tumors that arise from a delayed wound healing process. Among the central hub genes found in our study, we detected two carcinogenesisrelated genes. One of them, *HTR1A*, is a member of the 5-HT receptor superfamily that comprises six G-protein coupled receptor families and one ion channel family. *HTR1A* is widely involved in many human tumor types, including bladder, prostate, small cell lung, colorectal, and cholangiocarcinoma [17]. Highly selective blockers of 5-HT receptors have been used to determine the essential roles of *HTR1A*. It is implicated in cell proliferation in various types of cancer, such as bladder and prostate [18]. The pathway analysis in this study showed the involvement of serotonin signaling, which indicates that a 5-HT receptor blocker may be a therapeutic agent for the treatment of keloids. The other carcinogenesisrelated gene, *GNG13*, is a transduction factor for the G protein-coupled seven transmembrane helix receptors, which are associated with cancer development. The dysregulation of *GNG13* was implicated in the pathology of breast cancer [19] and an association between high *GNG13* expression and a malignant phenotype of gastrointestinal stromal tumor has been reported [20]. *GNG13* also was identified as a hub gene in PTEN-mutated prostate cancer [21]. The identification of *GNG13* and *HTR1A* as hub genes is consistent with keloids sharing some characteristics with cancer.

Misbalance between anti-angiogenic and proangiogenic growth factors, which, upon tumor cells transition to an angiongenic phenotype, leads to tumor growth beyond a defined size. Endostatin, periostin and vascular endothelial growth factor (VEGF) are associated with new vessel formation in the keloid lesions [22]. One of the over-expressed gene in the keloid samples, *NPY*, encodes a direct angiogenic stimulator that is known to stimulate cell proliferation. NPY-stimulated VEGF secretion and production contribute strongly to angiogenesis activity in human breast cancer [23], and it is also a promoter of prostate and breast cancers, influencing the proliferation and migration of cells [24]. In addition, *NPY* involves in the activation of hepatic stellate cells that contributes to hepatic cancer development [25].

Calcium influx has a vital role in keloid formation and dysregulation of Ca^2+^ ions in keloids has been documented [26]. Ca^2+^ channel blockers, such as Verapamil, have been administered to patients to reduce excessive extracellular matrix deposition. *ADCY8*, identified in our study, is an adenylate cyclase gene that encodes a membrane-bound enzyme that participates in cAMP formation [27]. Moreover, cAMP production is stimulated by calcium, which means that calcium catalyzes cAMP production through activation of *ADCY8*. Hence, *ADCY8* might mediate the effect of calcium ions on keloid fibroblasts.

Among the 349 predicted miRNAs and 378 TFs, hsa-mir-372, hsa-mir-20 and hsa-mir-10b had the highest degree in the constructed interaction with real hub genes, such as *GNG13, HTR1A* and *ADCY8*. Hsa-miR-372 has been identified as both a tumor promoter and a tumor suppressor, depending on the type of cancer [28]. One pro-tumorigenic mechanism of hsa-miR-372 is through de-repression of the tumor suppressor LATS2, while repression of IGF2BP1 by hsa-miR-372 is a tumor-suppressive mechanism [29, 30]. Hsa-mir-20 is involved in chronic wound healing, mainly through anti-angiogenic activity by targeting VEGF to inhibit the migration and proliferation of cells and tube formation [31]. Hsa-mir-10b is also involved in carcinogenesis via a negative feedback loop with TGF-β1, which indicates a possible role in keloid development [32]. SP1 as a potent inducer of extracellular matrix expression by fibroblasts [33], is a well-known TF involvement of keloid pathogenesis mainly by regulation of the extracellular matrix. However, a further investigation is still required to better comprehension of the underlying mechanisms in greater detail. In this study, we found that Sp1 interacted with *NPY* in the TF-DEG network.

Our results require experimental verification, which is demanding because of limited tissue samples. Small sample numbers can skew integrated bioinformatic analyses and important information might be ignored during the analysis. Because of the important effect of ethnicity on gene expression, the lack of sample classification during data processing and the ethnicity of the samples used in validation might also create bias in the results.

## Conclusion

*GNG13, ADCY8, NPY and HTR1A* may act as core genes in keloid formation and these core genes establish relationship with *SP1* and miRNA (hsa-mir-372, hsa-mir-20, hsa-mir-10*b*)*,* which may influence multiple signaling pathways in the pathogenesis of keloid.

## Supplementary Information

**Additional file 1.** degree.csv.

**Additional file 2.** mcc.csv.

**Additional file 3.** miR-target interaction.

**Additional file 4.** TF-target interatction.

## Data Availability

Microarray datasets (GSE7890, GSE44270, GSE92566 and GSE3189) for this study are openly available in Gene Expression Omnibus database at https://www.ncbi.nlm.nih.gov/geo/query/acc.cgi?acc=GSE7890, https://www.ncbi.nlm.nih.gov/geo/query/acc.cgi?acc=GSE44270, https://www.ncbi.nlm.nih.gov/geo/query/acc.cgi?acc=GSE92566, https://www.ncbi.nlm.nih.gov/geo/query/acc.cgi?acc=GSE3189, respectively.

## Abbreviations

NPY: Neuropeptide Y
HTR1A: 5-Hydroxytryptamine receptor 1A
SST: Somatostatin
ADCY8: Adenylate cyclase 8
NMUR1: Neuromedin U receptor 1
GNG3: G protein subunit gamma 3
GNG13: G protein subunit gamma 13
DEGs: Differentially expressed genes
PPI: Protein–protein interaction
GO: Gene ontology
TF: Transcription factor
